# Impairment of lysosomal quality control in Huntington disease

**DOI:** 10.1038/s41419-025-08103-z

**Published:** 2025-10-27

**Authors:** Paola Rusmini, Francesco Mina, Marta Valenza, Martina Vitali, Veronica Ferrari, Barbara Tedesco, Elena Casarotto, Marta Cozzi, Marta Chierichetti, Ali Mohamed, Paola Pramaggiore, Laura Cornaggia, Carmelo Milioto, Maria Brodnanova, Rocio Magdalena, Prashant Koshal, Margherita Piccolella, Riccardo Cristofani, Mariarita Galbiati, Valeria Crippa, Angelo Poletti

**Affiliations:** 1 Dipartimento di Scienze Farmacologiche e Biomolecolari “Rodolfo Paoletti”, Dipartimento di Eccellenza 2018-2027, Università degli Studi di Milano, Milan, Italy; 2 Dipartimento di Bioscienze, Università degli Studi di Milano, Milan, Italy

**Keywords:** Neurodegenerative diseases, Huntington's disease

## Abstract

Huntington disease (HD) is a neurodegenerative disease caused by a polyglutamine expansion (polyQ) in the Huntingtin protein (muHTT), which makes it prone to misfolding and aggregation. muHTT aggregates sequester a wide variety of proteins essential for cell homeostasis, including chaperones and transcription factors, and their depletion may contribute to HD pathogenesis. Lysosomes are the main hubs for degradative and signaling activities in cells, and their functionality is crucial for cell homeostasis, especially for neurons. Different forms of cellular stresses, including proteotoxic stresses, can alter lysosome integrity and induce lysosomal membrane permeabilization (LMP). Damaged lysosomes are recognized by galectins, in particular galectin-3 (LGALS3) with activation of the lysosome quality control (LQC) system responsible for repairing, degrading, or replacing leaky lysosomes. The system is transcriptionally regulated by the transcription factors EB and E3 (TFEB and TFE3, respectively). Using HD mouse and cell models, we demonstrated that TFEB and TFE3 are sequestered in muHTT aggregates, and muHTT proteins associates with LMP triggering the translocation of LGALS3 to the lumen of lysosomes, with a close relation between polyQ size and severity of these events. Moreover, we demonstrated that *TFEB* and *TFE3* silencing or overexpression modulate muHTT aggregation. TFEB and TFE3 knockdown worsens muHTT aggregation, while their overexpression reduces muHTT inclusions and concurrently reduces LGALS3 accumulation via lysophagy and lysosome replacement. Our findings suggest that both TFEB and TFE3 are implicated in HD, and their sequestration in muHTT inclusions increase the vulnerability of neurons to lysosome injury, altering LQC and contributing to disease pathogenesis.

**Figure:**
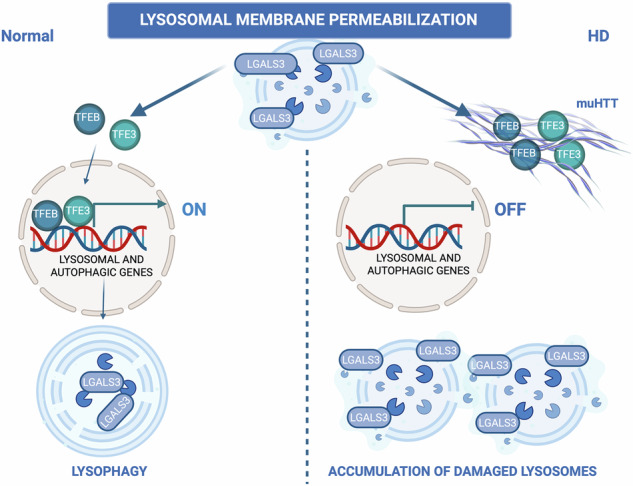
In physiologial conditions, lysosome membrane permeabilization occurs and activates TFEB and TFE3 triggering a response to induce lysophagy and lysosome biogenesis. In HD, muHTT sequesters TFEB and TFE3 into inclusions and the reduced TFEB/TFE3 bioavailability prevents the activation of lysophagy and leading to the accumulation of damaged lysosomes. Created in BioRender.

In physiologial conditions, lysosome membrane permeabilization occurs and activates TFEB and TFE3 triggering a response to induce lysophagy and lysosome biogenesis. In HD, muHTT sequesters TFEB and TFE3 into inclusions and the reduced TFEB/TFE3 bioavailability prevents the activation of lysophagy and leading to the accumulation of damaged lysosomes. Created in BioRender.

## Introduction

Huntington disease (HD) is a rare autosomal dominant inherited neurodegenerative disease (ND) characterized by both cognitive and motor alterations [1–3]. HD is caused by an expansion of the Cytosine, Adenine, Guanine (CAG) trinucleotide tandem repeat in the exon 1 of the *HTT* gene, located in the short arm of chromosome 4 [4, 5]. In healthy individuals, the CAG expansion ranges from 9 to 35 repeats, while 36 to 39 repeats may elicit the disease, and alleles with >40 repeats are pathogenic [6–8]. The expanded CAG repeat is translated into an elongated polyglutamine tract (polyQ) in the encoded mutant Huntingtin protein (muHTT). HTT is an evolutionarily conserved protein implicated in many physiological functions, acting as a scaffold for a number of binding proteins regulating multiple intracellular processes, including gene transcription, vesicular trafficking, and autophagy [9–11]. The polyQ tract located in muHTT causes protein misfolding and accumulation as amyloid aggregates, a hallmark of HD pathogenesis. The formation of soluble oligomers and the sequestration of essential proteins, including chaperones and transcription factors, contribute to muHTT proteotoxicity [12–15]. Moreover, impairment of the proteolytic systems, in particular of the autophagy-lysosomal pathway, has been observed in HD, contributing to disease pathogenesis [16–18].

Lysosomes are acidic organelles containing a large variety of hydrolases used to break down macromolecules through endocytosis, phagocytosis, or autophagy [19]. In addition to their role in autophagic clearance of protein aggregates (aggrephagy), lysosomes are also involved in the removal of macromolecules and damaged organelles. In particular, the quality control of organelles is ensured by organelle-specific autophagy, that selectively targets to clearance organelles such as the mitochondria (mitophagy), the endoplasmic reticulum (reticulophagy), and the lysosomes themselves (lysophagy) [20, 21]. Lysosomes also act as intracellular hubs for cell metabolism and nutrient sensing [22, 23], and they respond to stimuli by controlling and adjusting their position, motility, number, and size [24]. In this context, lysosomal functionality, positioning, and integrity are essential for maintaining cellular homeostasis, especially for neurons, postmitotic cells with high polarity and demands for metabolic activities.

Lysosomes are sensitive to membrane rupture and lysosomal membrane permeabilization (LMP), events that affect lysosomal integrity, increase permeability, and result in the release of luminal contents into the cytosol [25, 26]. In response to LMP, cells activate the lysosomal quality control (LQC), which includes lysosome repair, lysophagy, and regeneration by lysosomal biogenesis. Initially, LMP is recognized by galectins, in particular galectin-3/LGALS3, beta-galactoside-binding lectins that bind to the glycosylated luminal domain of lysosomal transmembrane proteins in the broken membrane [27–30]. In the early step of lysosomal injury or in presence of a small and limited lysosomal damage, LGALS3 recruits and activates the endosomal sorting complex required for transport (ESCRT) machinery for lysosomal repair [28, 31, 32]. Instead, if lysosomal repair fails or if there are large lysosomal ruptures, LGALS3 is responsible for the switching from ESCRT response to lysophagy, recruiting proteins involved in ubiquitination of lysosomal proteins and autophagy regulators. In the presence of lysosomal deficiency due to less efficient repair and/or lysophagy, a compensatory mechanism triggers the activation of transcription factors EB and E3 (TFEB and TFE3), which promotes lysosomal biogenesis and replacement [27, 28]. TFEB and TFE3 transcriptionally share the control of a set of genes involved in lysosomal functions and biogenesis by binding to the coordinated lysosomal expression and regulation consensus sequences, and similar molecular mechanisms regulate their activity. In HD, autophagy dysfunction together with impaired *TFEB* expression and TFEB activity were observed, and pharmacological or molecular approaches aimed at activating TFEB induced muHTT clearance reducing neurotoxicity [33, 34]. Notably, muHTT aggregates sequester TFEB through interaction with the prion-like domain (PrLD) present in the N-terminus of TFEB, while TFE3, lacking the PrLD, has not been detected in muHTT inclusions [35]. In addition, elevated levels of LGALS3 have been observed in the serum and brain of HD mice and patients [36], suggesting a role in the pathogenesis of the disease.

Here, we aimed to elucidate the contribution of TFEB, TFE3 and lysosomal damage to HD pathogenesis. In the presence of muHTT, we found that TFEB and TFE3 were sequestered in the inclusions, and that LGALS3 was recruited into damaged lysosomes. In addition, our investigation revealed that TFEB and TFE3 overexpression was capable of inducing both muHTT aggregates and damaged lysosome clearance restoring lysosomal functionality.

## Material and methods

### Plasmids and siRNAs

The HTT plasmids coding for the human *HTT-*exon1 carrying respectively 17, 73, 145 CAG repeats (HTTex1-17Q, HTTex1-73Q, HTTex1-145Q) were kindly provided by Prof. Manuela Basso (Department of Cellular, Computational and Integrative Biology, University of Trento, Italy). FLAG-TFEB and EGFP-TFEB plasmids were kindly provided by Prof. Andrea Ballabio (Telethon Institute of Genetics and Medicine (TIGEM), Dulbecco Telethon Institute, Federico II University, Naples). pEGFP-N1-TFE3 was a gift from Dr. Shawn Ferguson (Addgene plasmid #38120; http://n2t.net/addgene:38120; RRID:Addgene_38120). EGFP-LGALS3 plasmid was kindly provided by Prof. Marja Jäättelä (Danish Cancer Society Research Center, Copenhagen, Denmark). TFE3 plasmid was obtained by excising the EGFP sequence from pEGFP-N1-TFE3 with KpnI/NotI and inserting an in-frame sequence including a STOP codon (5ʹ GAT TCA GCG GCC GCC AGG GGA GGG GTG AGGCCT GAT CAT GGT ACC GTC TAA GT 3ʹ). The following siRNA duplex was used for silencing *Tfeb* expression: siRNA sense: 5ʹ GGAUCAAGGAGCUGGGAAUUU 3ʹ, antisense: 5ʹ AUUCCCAGCUCCUUGAUCCUU 3ʹ (Dharmacon); *Tfe3* expression: siRNA sense: 5ʹ GCAAAUUACAGAAGGAACAUU 3ʹ, antisense: 5ʹ UGUUCCUUCUGUAAUUUGCUU 3ʹ; the non-targeting siRNA sense: 5ʹ UAGCGACUAAACACAUCAAUU 3ʹ and antisense: 5ʹ UUGA UGUGUUUAGUCGCUAUU 3ʹ (Dharmacon).

### Cell cultures and transfections

Neuroblastoma X spinal cord (NSC-34) cells are mouse motoneuron immortalized cells routinely used in our laboratory [37–41]. NSC-34 cells were maintained in Dulbecco’s modified Eagle’s medium High Glucose (EuroClone, Pero, Italy, ECB7501) supplemented with 1 mM L-Glutamine (EuroClone, ECB3004D), penicillin-streptomycin (Euroclone, ECB3001D) and 5% fetal bovine serum non-USA origin (Sigma-Aldrich, F7524) at 37 °C in 5% CO_2_.

The immortalized mouse embryonic striatal knock-in cell line (parental cell line ST-Kin HDH Q7/7) [42] was maintained at the permissive temperature of 33 °C in Dulbecco’s modified Eagle’s medium High Glucose, supplemented with 10% fetal bovine serum (GIBCO, A4766801), 2 mM L-glutamine (EuroClone, ECB3004D), and penicillin-streptomycin. NSC-34 and ST cells were transfected with 1 μg/mL and 1.5 μg/mL of DNA plasmid respectively, using Lipofectamine 3000^®^ Transfection Reagent (Thermo Fisher Scientific, Waltham, MA, USAL3000-015) following the manufacturer’s protocol. siRNAs were transfected for 72 h using Lipofectamine3000 with 40 pmol in 12-well plates and 20 pmol in 24-well plates.

### Animals

Two different HD colonies were used in this study: R6/2 colony and zQ175DN colony. R6/2 mice (RRID:IMSR_JAX: 002810) are transgenic mice expressing exon 1 of the human mutant huntingtin with 160 +/-5 CAG [43, 44]. These mice exhibit early and rapid progression of behavioral, molecular, and electrophysiological abnormalities, starting at 6 weeks of age and die at around 13 weeks of age. Brains from R6/2 and relative control mice were kindly provided by Laura Colombo and Mario Salmona (Mario Negri Institute, *authorization n. 786/2021-PR*). The zQ175 neo-deleted knock-in mice (zQ175DN mice) have the mouse *Htt* exon 1 replaced by the human *HTT* exon 1 sequence with a ~190 CAG repeat tract. These mice show motor deficits starting from 9 months, while clinical signs and survival deficits appear at 12 months of age. The zQ175DN mice were acquired by The Jackson Laboratories (B6.129S1-*Htt*^*tm1.1Mfc*^/190ChdiJ; RRID:IMSR JAK:029928) and maintained by mating male and female heterozygous mice with C57BL6/J mice (purchased from Charles River). Mice were weaned and then genotyped at 3 weeks of age (±3 days). Mice were housed under standard conditions in enriched cage (22 ± 1 ^◦^C, 60% relative humidity, 12 h light/dark schedule, 3–4 mice/cage, with food and water ad libitum). All animal experiments were approved and carried out in accordance with Italian Governing Law (D. lgs 26/2014; Authorization *n.017/2024-PR*).

### Immunofluorescence in brain slices

HD and age-matched wild-type mice were anesthetized by intraperitoneal injection of 2.5% Avertin (250 mg/kg body weight) and transcardially perfused with saline solution followed by 4% paraformaldehyde (PFA). Brains were post-fixed in 4% PFA at 4 °C overnight and then incubated in 30% sucrose to prevent ice crystal damage during freezing in Optimal Cutting Temperature compound (OCT). Immunofluorescence (IF) staining was performed on 15 μm coronal sections. Epitopes were demasked at 98 °C with NaCitrate (10 mM, pH 6) for 15 min. Tissue slices were then blocked and permeabilized with 5% Normal Goat Serum (NGS) and 0.5% Triton for 1 h at room temperature (RT), followed by incubation with rabbit anti-TFEB antibody (1:100; Fortis Life Sciences, cat. A303-673A) or rabbit anti-TFE3 antibody (1:100; Merck-Sigma Aldrich cat. HPA023881) and with mouse anti-muHTT antibody (1:100; Merck Millipore, cat. MAB5374) diluted in phosphate buffered saline (PBS) + 0.1% Triton and 1% NGS for 3 h at RT. Slices were washed 3 times (10 min each) with PBS + 0.1% Triton at RT and exposed to anti-mouse Alexa Fluor 488-conjugated goat secondary antibody (1:500; Invitrogen) and to anti-rabbit Alexa Fluor 568-conjugated goat secondary antibody (1:500; Invitrogen) for 1 h at RT. Sections were counterstained with the nuclear dye Hoechst 33258 (1:10,000, Invitrogen), washed 3 times (10 min each) with PBS + 0.1% Triton at RT and mounted under coverslips using VECTASHIELD® Antifade Mounting Medium (# H-1000-10, Vector Laboratories).

Images were acquired with a Nikon Eclipse Ti2-E spinning disk confocal microscope (NoLimits Facility, Università degli Studi di Milano, Italy). Laser intensity and detector gain were maintained constant for all images and z-stacks (step size of 0.33 μm; z-vol 5 μm) images were acquired at 60x. Deconvolution (Fiji) was applied to the images. Crops around single cells were done and shown as representative images to appreciate the co-localization with muHTT aggregates.

### Reverse transcription and quantitative polymerase chain reaction

For quantitative polymerase chain reaction (q-PCR), ST cells were seeded at 260,000 cell/well in 6-well plates; NSC-34 were seeded at 180,000 cells/well in 6-well plates. Total RNA was extracted 48 h after transfection, using Tri-Reagent (Merck, T9424) following the manufacturer’s protocol. For mouse tissue, frozen tissues were homogenized with Tissue-Lyser II and stainless steel glass beads (Qiagen, Hilden, Germany) in TRI Reagent, and RNA was extracted and purified with Direct-zol RNA miniprep Plus kit (Zymo Research, R2072).

RNA concentration and quality were tested measuring the ratio of the absorbance at 260/280 nm and 260/230 nm for each sample using NanoDrop 2000 (Thermo Fisher Scientific, ND-2000). 1 μg per sample was treated with DNase (Sigma-Aldrich, AMPD1) and reverse transcribed using the High-Capacity cDNA Reverse Transcription Kit (Thermo Fisher Scientific, 4368814). q-PCR was performed using the CFX96 Real-Time System (Bio-Rad Laboratories), the iTaq SYBR Green Supermix (Bio-Rad Laboratories, 1725124), and with a final concentration of 0.5 μM of primers. The primer sequences are listed in Table S1. Data were normalised using *Rplp0* gene. The experiments were performed with 4 independent samples (*n* = 4).

### Western blotting and filter retardation assay

For western blotting (WB) and filter retardation assay (FRA), ST-Kin 7/7Q were seeded at 130,000 cells/well in 12-well plates; NSC-34 were seeded at 90,000 cells/well in 12-well plates. Protein extracts were prepared as described in ref. [45] and quantified with the bicinchoninic acid method using QPRO-BCA kit Standard (PRTD1, Cyanagen, Bologna, Italy). For WB analysis, 15 μg of total proteins were loaded on 10% (or 12%) sodium dodecyl sulfate-polyacrylamide gels. After electrophoresis, the gels were electro-transferred to a nitrocellulose membrane by performing an overnight electrophoretic transfer in a tank transfer system. For FRA, 20 μg of total proteins were filtered through a 0.22-μm cellulose acetate membrane (Whatman, 100404180) using a Bio-Dot SF Microfiltration Apparatus (Bio-Rad Laboratories, 1703938).

To perform soluble/insoluble fractionation, the protocol described in ref. [46] was used. The samples were then analyzed by WB, loading 5 μg of total proteins for each fraction on 10% sodium dodecyl sulfate-polyacrylamide gels. The membranes obtained by WB or FRA were incubated with primary antibodies diluted in blocking solution (5% non-fat dried milk (Euroclone, EMR180500) in TBS-T (20 mM Tris base, 140 mM NaCl pH 7.6, and 0.01% Tween 20 [Sigma-Aldrich, P1379])). Immunoreactivity was detected after incubation for 1 h with peroxidase-conjugated secondary antibodies with enhanced chemiluminescent detection reagent (Westar Antares, Cyanagen, Bologna, Italy; XLS0142). Images were acquired with a Chemidoc XRS System (Bio-Rad Laboratories, 1708265).

Primary and secondary antibodies are listed in Table S2.

Densitometric quantification analysis was performed using Image Lab Software, version 5.2.1 (Bio-Rad Laboratories, Hercules, CA, USA).

### Immunofluorescence analysis

For IF analysis, ST KIN 7/7Q cells were seeded at 60,000 cells/well on 13-mm coverslips coated with Poly-L-ornithine hydrobromide (Merck, P3655). NSC-34 were seeded at 30,000 cells/well in 24-well plates on 13-mm coverslips. Transfection/treatments were performed as previously explained in the text. The cells were fixed with 4% PFA in PBS 48 h after transfection and then permeabilized in 0.2% Triton X-100 in PBS for 20 min. Cells were incubated overnight with primary antibodies previously listed in Table S2 in blocking solution and then for 1 h with secondary antibody goat Alexa Fluor® 488 or Alexa Fluor® 594 in blocking solution. Nuclei were stained with 4’,6-diamidino-2-phenylindole (DAPI) (Sigma-Aldrich, D4592, 0.01%in PBS). The coverslips were mounted with Mowiol^®^ 4-88 (Merck-Millipore, 475904) and analyzed with LSM900 confocal microscope Meta system (Zeiss, Germany). Images were processed with the ZEN microscopy software (Zeiss).

### LGALS3 puncta assay

Cells were seeded, co-transfected with plasmids encoding EGFP-LGALS3 and the HTT proteins and then processed as described for IF assay. A manual quantification of cells with >3 EGFP-LGALS3 puncta was carried out in 3 randomly selected visual fields per sample, using a PL 40x eyepiece with focal grid (100 × 10 mm in 100-grid divisions), as previously described [38, 47]. The analysis included 3 samples per condition and at least 100 cells for each condition were counted (*n* = 3).

### MTT cell viability assay

The MTT assay (3-(4,5-dimethylthiazolyl-2)-2,5-diphenyltetrazolium bromide, Merck, M5655) was used to assess cell metabolic activity and viability. NSC-34 cells were seeded at a density of 45,000 cells per well in 24-well plates and transfected as described in the text. Following 48 h of treatment, the medium was removed, and cells were incubated with 300 µL of MTT solution at 37 °C for 30 min. Subsequently, 500 µL of 2-propanol was added to each well to solubilize the formazan crystals. Absorbance was recorded at 570 nm using an Enspire® Multimode Plate Reader (PerkinElmer, Waltham, MA, USA). All experiments were performed with six independent replicates (*n* = 6).

### Flow cytometric analysis of inclusions and trafficking

For flow cytometric analysis of inclusions and trafficking (FLoIT), NSC-34 cells were seeded at 30,000 cells/well in 24-well plates. Transfection/treatments were performed as previously described in the text. 48 h after transfection, cells were harvested and resuspended in 300 µl of PBS without Ca^2+^ and Mg^2+^ with 5% FBS. Cell suspensions were divided into 2 aliquots (150 µl each): one was supplemented with DRAQ7 (Thermo Fisher, D15106), a membrane-impermeable fluorescent DNA dye, while the other aliquot was treated with lysis buffer (PBS w/o Ca^2+^ and Mg^2+^, 1% Triton X-100, and 1% protease inhibitors cocktail), supplemented with 0.002% DAPI, with the exception of the control samples used to set the gates. Samples were incubated in ice for 2 min, and flow-cytometry analysis was carried out using a Novocyte 3000 flow cytometer (ACEA biosciences) and NovoExpress software (version 1.4.1; ACEA biosciences). Exclusion of debris and photomultiplier (PMT) noise was achieved setting the forward scatter (FSC) threshold at 100,000. Analysis was performed by flow cytometry using forward and side scatter, DRAQ7 fluorescence (640 nm excitation, 660/20 nm emission), DAPI fluorescence (405 nm excitation, 445/45 nm emission), and GFP fluorescence (488 nm excitation, 530/30 nm emission). Non-lysed samples were used to exclude non-viable cells (stained with DRAQ7) and to determine transfection efficiency. PMT voltage was set to 540 V (DRAQ7) and 373 V (GFP). For the lysed samples the FSC threshold was set to 1000 (corresponding to standard microsphere diameter <2 mm, as reported in the NovoCyte^®^ flow cytometer operator’s guide) to include small inclusions in the analysis. All axes were set to log10. PMT voltage was set to 482 V (GFP) and 501 V (DAPI). Non-transfected samples were used as negative control, to set the GFP+ threshold. Nuclei were detected by FSC-A parameter and DAPI fluorescence. Cytoplasmatic events were analyzed using GFP fluorescence and FSC-H parameter. Inclusion size was calculated using FSC-H, and inclusion number per transfected cells was calculated as described by Whiten et al. [48].

### Statistical analysis

Data are shown as mean ± SD unless otherwise specified. Statistical analysis was performed with analysis of variance (ANOVA): the effect of one independent variable was compared using a One-Way ANOVA test; the effect of two variables was compared by a Two-Way ANOVA test. A *P* value < 0.05 was considered statistically significant. When ANOVA was significant, we performed a post hoc test (see Figure legends for details). Analyzes were carried out using the PRISM (version 9) software (GraphPad Software).

## Results

### TFEB and TFE3 are sequestered into muHTT inclusions in the HD mouse brain

Based on the evidence that proteostasis is highly impaired in HD and that autophagy is involved in muHTT clearance, we started to investigate the role of the main regulators of lysosomal function and autophagy, TFEB and TFE3, in HD. As disease models, we used the transgenic R6/2 mice, overexpressing exon 1 of human muHTT [49], and the zQ175DN (delta-neo) heterozygous (het) knock-in mice, expressing a CAG repeat stretch of 190 into the mouse huntingtin gene [43, 44].

**Fig. 1:**
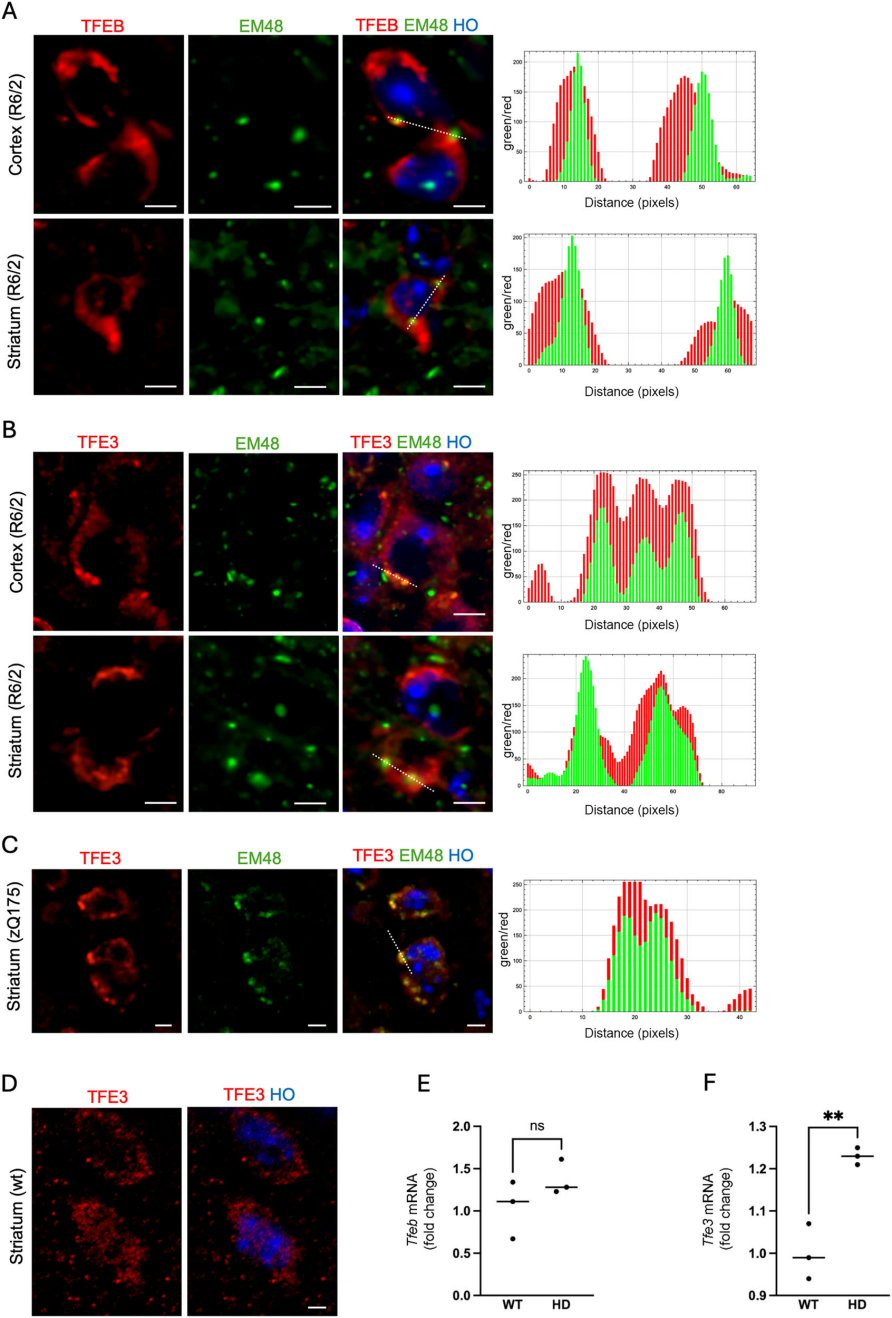
TFEB and TFE3 co-localized with muHTT aggregates in the mouse HD brains. Representative confocal images of the immunolabeling of TFEB (**A**) or TFE3 (**B**) (red) and muHTT aggregates positive for EM48 antibody (green) in cortex and striatum of R6/2 mice at 12 weeks of age (*n* = 3/genotype), and of TFE3 in striatum of zQ175DN mice at 30 weeks of age (**C**). The relative Plot Profile analysis (ImageJ) was performed in **A**, **B**, **C** to graphically visualize the co-localization of TFEB or TFE3 with muHTT aggregates. **D** Representative confocal image of the immunolabeling of TFE3 (red) in the striatum of wt mice. Hoechst (HO, blue) was used to counterstain nuclei in (**A**–**D**). Crops for single cells (in **A**–**D**) were done from the original images (60X magnification) to appreciate the co-localization. Scale bar in (**A**–**D**): 5 μm. **E**, **F** q-PCR performed in the striatum of wt and R6/2 mice (*n* = 3/group). Data are expressed as mean and individual values for 3 independent samples (**p < 0.005, unpaired *t* test).

We first tested the localization of TFEB and TFE3 in the presence of muHTT aggregates in HD mouse brains. The IF staining with antibodies against these transcription factors in combination with the EM48 antibody, which specifically recognizes different forms of muHTT, showed that both TFEB and TFE3 were sequestered into muHTT aggregates in the striatum and cortex of R6/2 mice (Figs. 1A, B and S1). We also tested TFE3 localization in the zQ175DN mouse brain. As shown in Fig. 1C, we confirmed the sequestration of TFE3 into muHTT aggregates also in this HD mouse model. Moreover, we found that TFE3 showed a more diffused pattern in wild-type (wt) brains compared to HD brains (Figs. 1D and S2), supporting the hypothesis that its sequestration occurs only in the presence of muHTT aggregates. Notably, q-PCR showed a slight but significant upregulation of *Tfe3* (Fig. 1F) expression in the striatum of HD mice, while *Tfeb* expression levels remained unchanged (Fig. 1E).

Collectively, these findings suggest that not only TFEB, but also TFE3 can be involved in HD pathogenesis in vivo in HD mice.

### TFEB and TFE3 are sequestered into muHTT aggregates in HD cell models

**Fig. 2:**
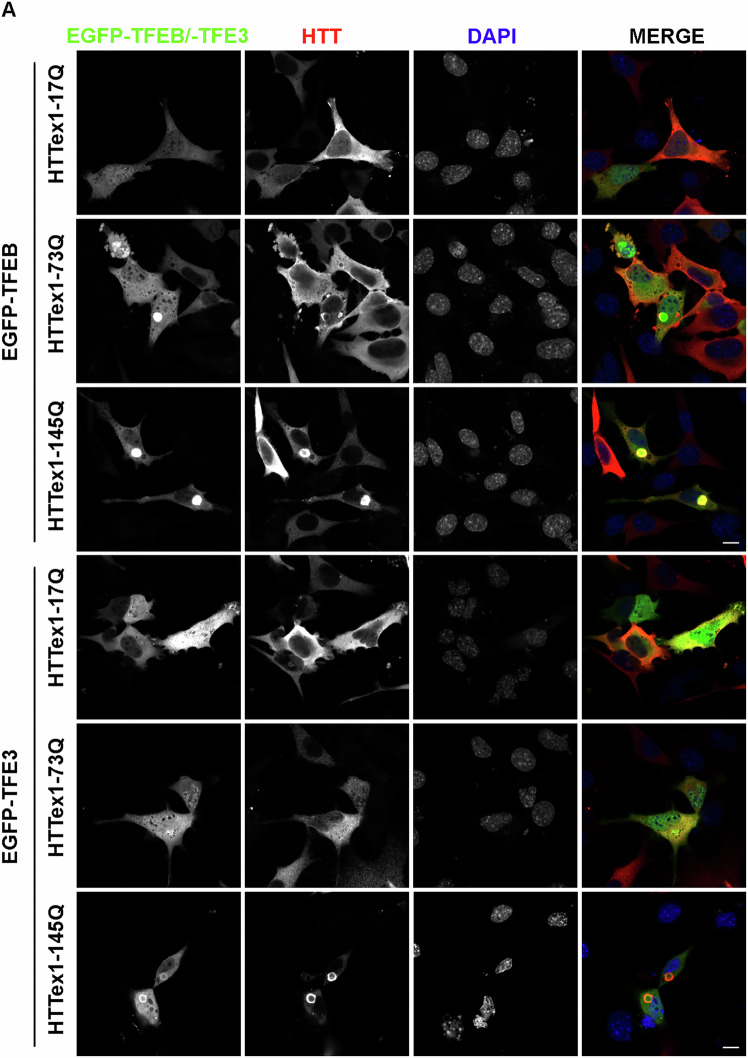
TFEB and TFE3 accumulated into muHTT aggregates in ST cells. ST cells were co-transfected with wt or muHTT and EGFP-TFEB or EGFP-TFE3. **A** Representative images by confocal microscopy of EGFP-TFEB and IF staining with anti-HTT antibody (red), nuclei were stained with DAPI (blue) (63X magnification). Scale bar: 10 μm.

To better extend our in vivo analysis, we designed a novel approach based on a reconstituted HD cell model. Using the parental immortalized striatal knock-in cell line ST KIN, we overexpressed wt (HTTex1-17Q) or mutant exon-1 *HTT* (muHTT) with different polyQ lengths (HTTex1-73Q or HTTex1-145Q) in the presence of EGFP-TFEB or EGFP-TFE3. By IF analysis, we observed that GFP-TFEB and GFP-TFE3 showed a diffuse distribution into the cells in presence of wtHTT. Conversely, muHTT formed inclusions that sequestered both EGFP-TFEB and EGFP-TFE3 (Fig. 2), recapitulating the in vivo findings.

**Fig. 3:**
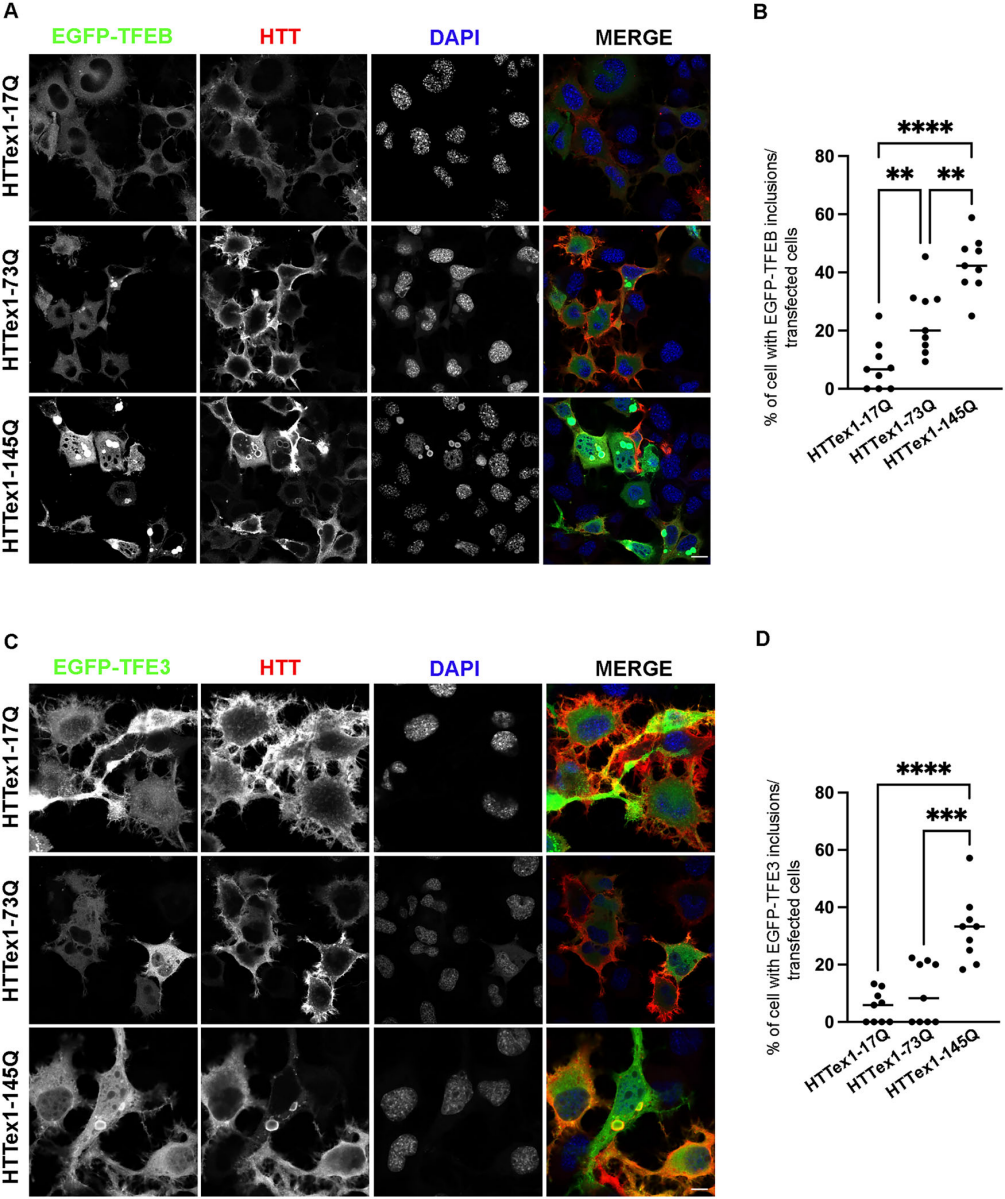
EGFP-TFEB and EGFP-TFE3 are sequestered in muHTT aggregates in an in vitro HD cell model. **A**–**D** NSC-34 cells were co-transfected with wt or muHTT and EGFP-TFEB or EGFP-TFE3. **A** Representative images by confocal microscopy of EGFP-TFEB and IF staining with anti-HTT antibody (red), nuclei were stained with DAPI (blue) (63X magnification). Scale bar: 10 μm. **B** The graph with individual values represents the percentage of cells with EGFP-TFEB inclusions among total GFP-positive cells; the fields were randomly selected and at least 100 cells for each sample were analyzed (*n* = 3) (**p < 0.005, ***p < 0.001, ****p < 0.0001, one-way ANOVA with Tukey’s test). **C** Representative images by confocal microscopy of EGFP-TFE3 and IF staining with anti-HTT antibody (red), nuclei were stained with DAPI (blue) (63X magnification). Scale bar: 10 μm. **D** The graph with individual values represents the percentage of the cells with EGFP-TFE3 inclusions on total GFP-positive cells; the fields were randomly selected and at least 100 cells for each sample were analyzed (*n* = 3) (***p < 0.001, ****p < 0.0001, one-way ANOVA with Tukey’s test).

**Fig. 4:**
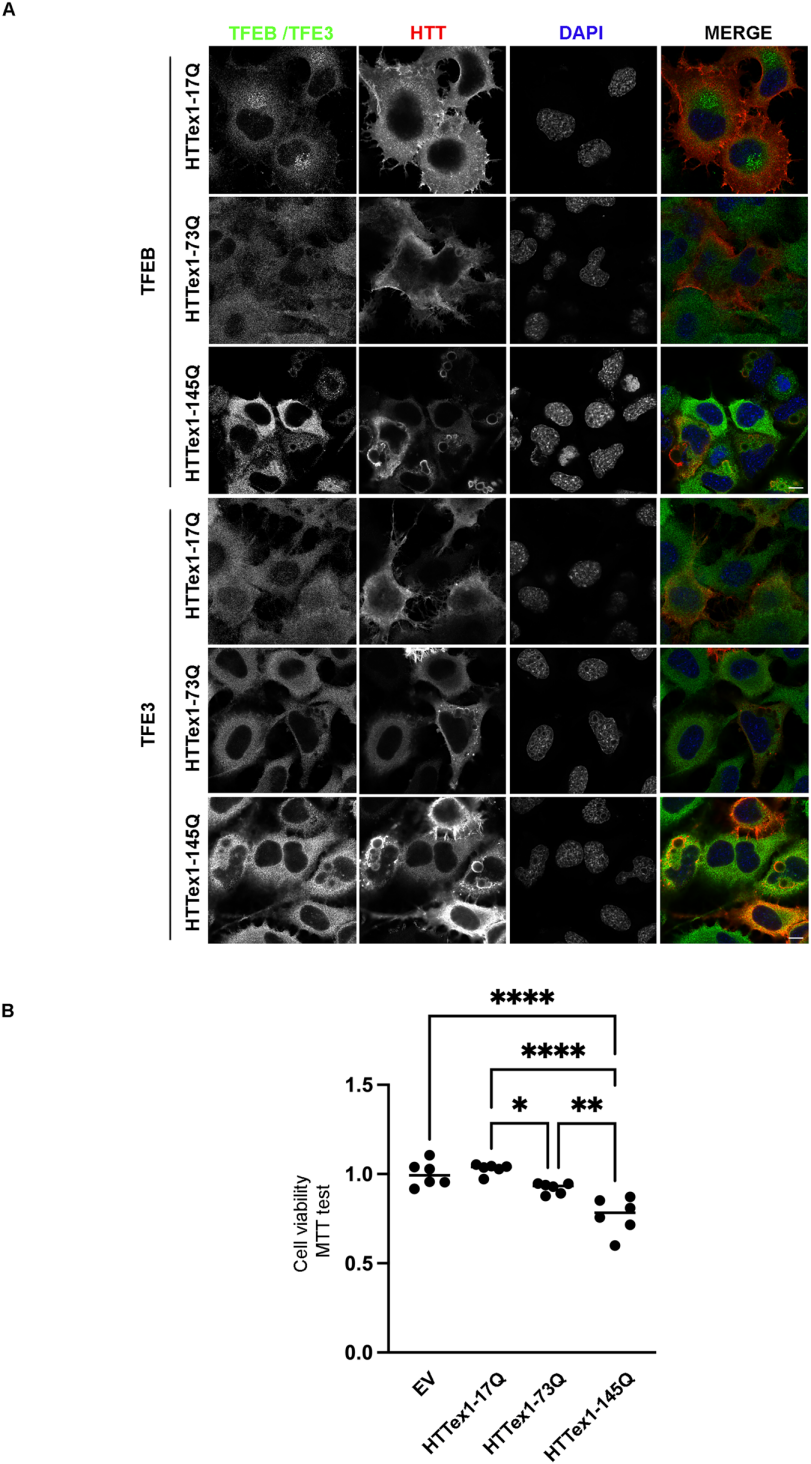
Endogenous TFEB and TFE3 are sequestered into muHTT aggregates in an in vitro HD cell model. **A** NSC-34 cells were transfected with wt or mutant HTT. Representative images by confocal microscopy of double immunostaining with anti-TFEB or anti-TFE3 (green) and anti-HTT antibody (red), nuclei were stained with DAPI (blue) (63X magnification). Scale bar: 10 μm. **B** NSC-34 cells were transfected with wt or muHTT and pCDNA3 as empty vector. The graph with individual values represents the cell viability for *n* = 6 independent samples (*p < 0.05, (** *p* < 0.005, ****p < 0.0001, one-way ANOVA with Tukey’s test).

To confirm these data and to elucidate the involvement of TFEB and TFE3 in HD, we exploited a second neuronal cell line, the NSC-34 cells [38]. We overexpressed wtHTT or muHTT with EGFP-TFEB or EGFP-TFE3 and found that, similarly to striatal cells, muHTT inclusions sequestered both EGFP-TFEB and EGFP-TFE3 in NSC-34 cells. Interestingly, EGFP-TFEB inclusions were present at comparable levels both in 73Q and 145Q muHTT-over-expressing cells (Fig. 3A, B), while EGFP-TFE3 inclusions were significantly increased only in the presence of HTTex1-145Q inclusions compared to wt and 73Q (Fig. 3C, D). Due to the propensity of GFP proteins to oligomerize and aggregate when used in some chimeric proteins, we performed the analysis on the endogenous forms of TFEB and TFE3. The results confirmed that both the endogenous TFEB and TFE3 colocalized with muHTT inclusions, while in the presence of HTTex1-17Q, both transcription factors displayed a diffuse distribution in the cells (Fig. 4A). MuHTT expression in these cells led to impaired cell viability and toxicity correlated with polyQ length tract (Fig. 4B).

**Fig. 5:**
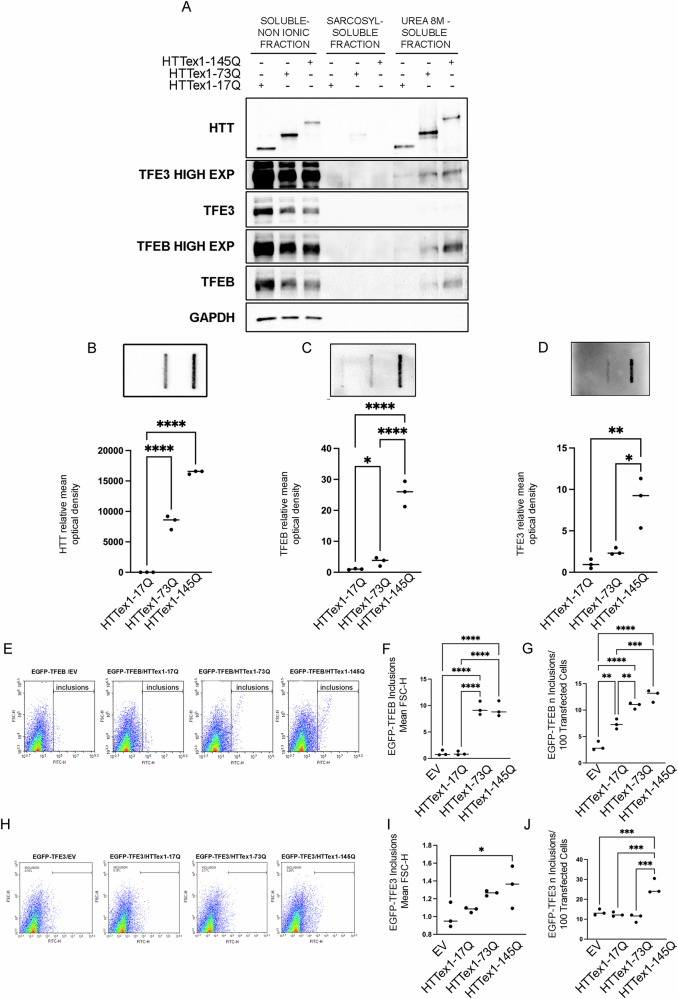
Analysis of TFEB and TFE3 aggregates. **A**–**D** NSC-34 cells were transfected with wt or muHTT **A** WB analysis of soluble and insoluble protein fractions was performed, GAPDH was used for loading control. FRA was performed to detect muHTT (**B**), TFEB (**C**), and TFE3 (**D**) high-molecular species. The graphs with individual values represent the mean relative optical density of FRA for *n* = 3 independent samples (* *p* < 0.05, **p < 0.005, ****p < 0.0001, one-way ANOVA with Tukey’s test). **E**–**J** FloIT analysis on NSC-34 cells co-transfected with wt or muHTT and EGFP-TFEB (**E**) or EGFP-TFE3 (**H**). The bar graphs with individual values represent the inclusions size (**F**, **I**) and the ratio of number of inclusions to 100 transfected cells (**G**, **J**) for *n* = 3 independent samples (*p < 0.05, **p < 0.005, ***p < 0.001, ****p < 0.0001, one-way ANOVA with Tukey’s test).

muHTT is present in different forms in the cells: soluble monomers, oligomeric forms, and insoluble inclusions [12, 50]. To gain further insight into the biochemical properties of TFEB and TFE3 sequestration into muHTT inclusions, we performed a fractionation assay to isolate and analyze soluble and insoluble protein fractions. By WB analysis, we observed that wt and muHTTs were present both as soluble proteins in the soluble non-ionic fraction and as inclusions in the urea-soluble fraction. TFEB and TFE3 were both present in the soluble non-ionic fraction in all the samples; interestingly, they were detected in the urea fractions only in HTTex1-73Q and HTTex1-145Q samples (Fig. 5A).

The data were validated by FRA, in which cell lysates are filtered through a cellulose acetate membrane to retain the high-molecular weight species, while soluble and monomeric forms pass through the filter unretained. FRA analyses and quantifications showed that only muHTTs (HTTex1-73Q and HTTex1-145Q) were retained on the cellulose acetate membranes, conversely HTTex1-17Q passed through the membrane (Fig. 5B). In the presence of muHTT, both endogenous TFEB and TFE3 were retained on the membranes, confirming the sequestration into high-molecular weight species (Fig. 5C, D). TFEB was significantly retained on the cellulose acetate membrane both in presence of HTTex1-73Q and of HTTex1-145Q, while TFE3 accumulation was significantly observed only in presence of HTTex1-145Q.

The intracellular accumulation of TFEB/TFE3 was also analyzed by FloIT, a sensitive technique capable of resolving inclusions based on their different size and protein composition [48, 51]. Briefly, NSC-34 cells were co-transfected with EGFP-TFEB/TFE3 plus wt/muHTTs (Fig. 5E–J) and we observed that, in the presence of HTTex1-73Q and HTTex-145Q, the size and number of EGFP-TFEB inclusions were significantly increased compared to control (Fig. 5E–G). Instead, only HTTex-145Q was able to induce an increase in the number of EGFP-TFE3 inclusions, even if the size of these inclusions remained unchanged (Fig. 5H–J).

### TFEB and TFE3 are involved in the clearance of muHTT

It is known that TFEB is dysregulated in HD, and that its overexpression can boost the autophagic clearance of muHTT, reducing its accumulation into inclusions [33, 52, 53]. The role of TFE3 in HD has not yet been elucidated. Hence, modulating *TFEB* and *TFE3* expression through gene silencing or overexpression, we evaluated the involvement of TFEB and TFE3 in the clearance of muHTT.

**Fig. 6:**
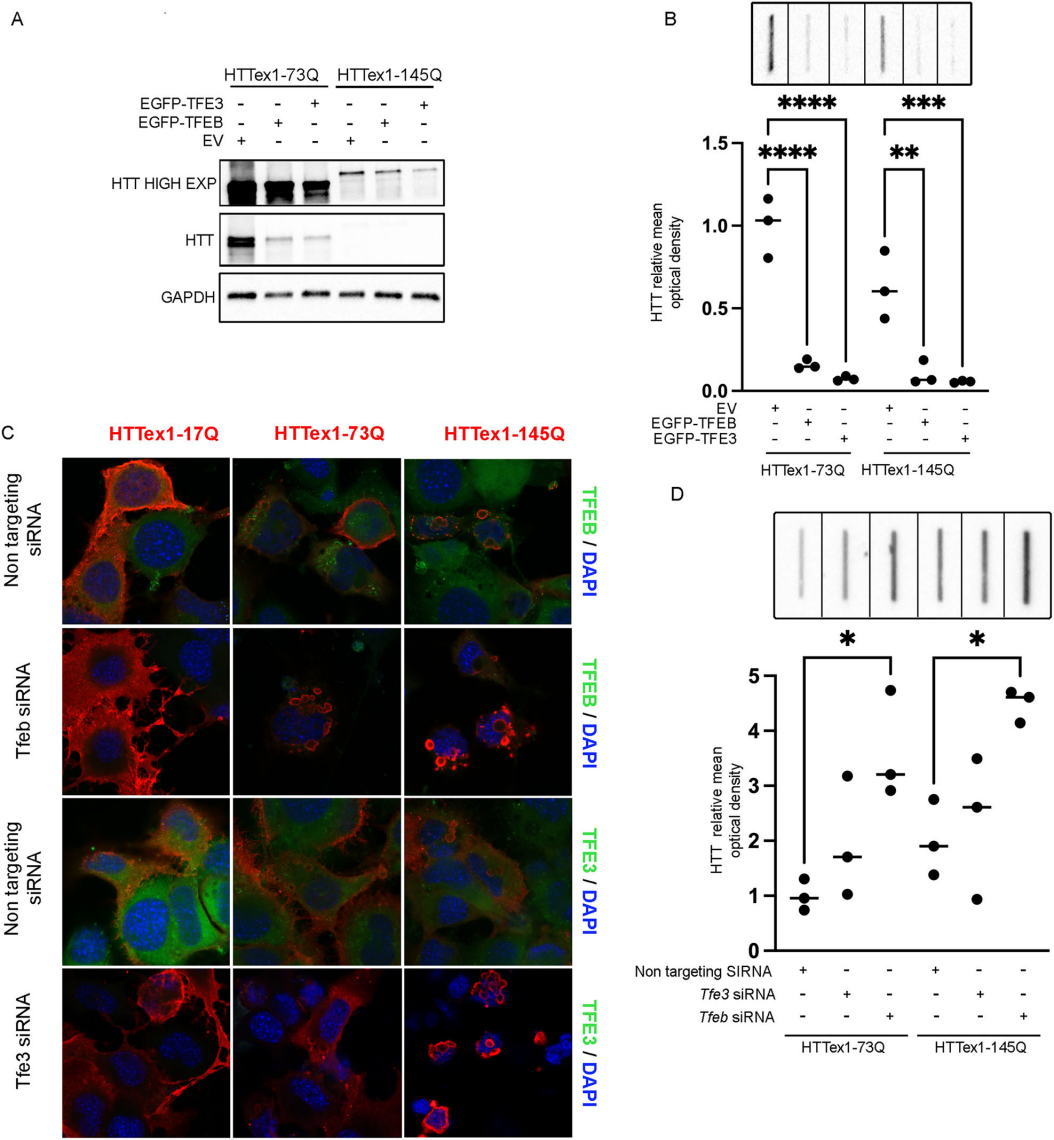
*Tfeb/Tfe3* silencing or overexpression modulates muHTT aggregation and lysosomal functionality. **A**, **B** NSC-34 cells were co-transfected with EGFP-TFEB, EGFP-TFE3, or empty-vector (EV) and muHTT. **A** WB analysis and **B** FRA were performed. The graph with individual values represents the mean relative optical density of FRA for *n* = 3 independent samples (**p < 0.005, ***p < 0.001, ****p < 0.0001, two-way ANOVA with Bonferroni’s test). **C**, **D** NSC-34 cells were transfected with *Tfeb, Tfe3* or non-targeting (as control) siRNAs and wt or mutant *HTT*. **C** Representative images by confocal microscopy of double immunostaining with anti-TFEB or anti-TFE3 (green) and anti-HTT antibody (red), nuclei were stained with DAPI (blue) (63X magnification). Scale bar: 10 μm. **D** FRA was performed, and the graph with individual values represents the mean relative optical density of FRA for *n* = 3 independent samples. (*p < 0.05, two-way ANOVA with Bonferroni’s test).

By WB and FRA, we observed that both *TFEB* and *TFE3* overexpression was capable of clearing the muHTT soluble and insoluble forms (Fig. 6A, B), and conversely both *TFEB* and *TFE3* silencing increased muHTT accumulation, but only *TFEB* downregulation significantly worsened muHTT aggregation (Fig. 6C, D).

These data suggest that TFEB and TFE3 play different roles in the clearance of muHTT.

### TFEB and TFE3 sequestration affects the lysosomal functionality

To understand the effects of TFEB and TFE3 sequestration into muHTT inclusions and due to their crucial role in the regulation of autophagy, lysosomal function and biogenesis, we evaluated the expression of genes controlled by TFEB/TFE3 and involved in the proteostasis pathways in presence of wt and muHTTs.

**Fig. 7:**
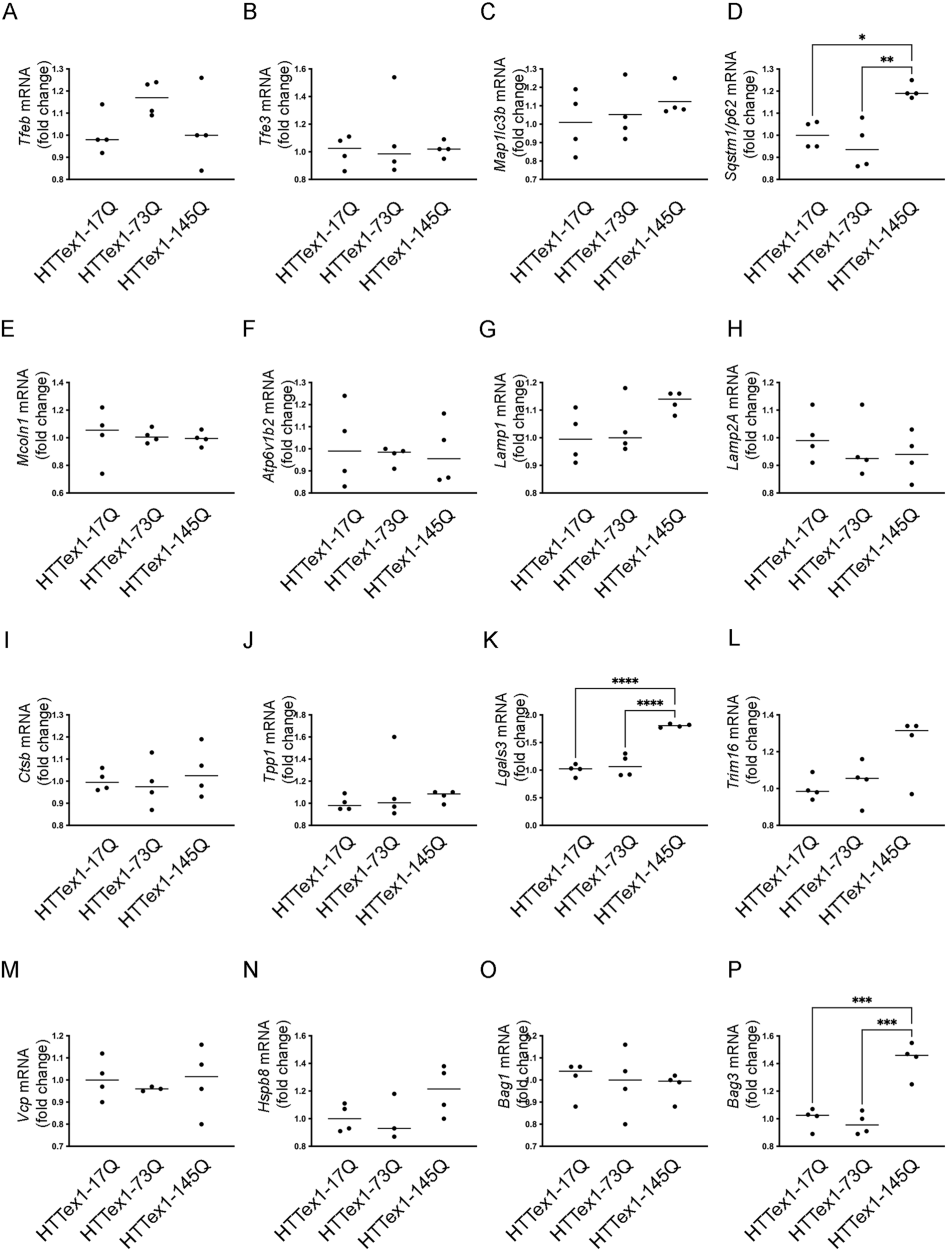
Effects of TFEB and TFE3 sequestration into muHTT aggregates on lysosomal and autophagic genes expression. q-PCR was performed on NSC-34 cells transfected with wt or muHTT. The relative fold difference in mRNA expression was determined using HTTex1-17Q as internal control. Data are with individual values with means of 4 independent samples. q-PCR on the following mRNA: *Tfeb* (**A**); *Tfe3* (**B**); *Map1lc3b* (**C**); *Sqstm1/p62* (**D**); *Mcoln1* (**E**); *Atp6v1b2* (**F**); *Lamp1* (**G**); *Lamp2a* (**H**); *Ctsb* (**I**); *Tpp1* (**J**); *LGals3* (**K**); *Trim16* (**L**); *Vcp* (**M**); *Hspb8* (**N**); *Bag1* (**O**); *Bag3* (**P**). The graphs represent the relative fold induction of these genes (*p < 0.05, **p < 0.005, ***p < 0.001, ****p < 0.0001, one-way ANOVA with Tukey’s test).

First, we assessed *Tfeb* and *Tfe3* (Fig. 7A, B) expression and, unlike the data obtained in mouse brain, no variations were observed in the three experimental groups (HTTex1-17Q, HTTex1-73Q, and HTTex1-145Q). Then, we analyzed the expression of relevant autophagic genes, like microtubule associated protein 1 light chain 3 beta (*Map1lc3b), Sequestosome1/p62 (Sqstm1/p62)* (Fig. 7C, D), and found that, in presence of HTTex1-145Q a slight increase of *Sqstm1/p62* expression is detectable; this suggests an involvement of this important gene coding for the selective autophagy receptor SQSTM1/P62, which is generally upregulated in presence of proteotoxic stress [54].

No variation was observed in the expression of lysosomal genes coding for transmembrane proteins mucolipin TRP cation channel 1 (*Mcoln1)*, ATPase H+ transporting V1 subunit B2 (Atp6v1b2), lysosomal associated membrane protein (Lamp1), and *Lamp2A* (Fig. 7E–H), and for luminal enzymes catepsin b (*Ctsb)* and tripeptidyl-peptidase 1 (Tpp1) (Fig. 7I, J). Next, we analyzed the expression of genes involved in the LQC: *Lgals3*, involved in the recognition of lysosomal damage (Fig. 7K); tripartite motif containing 16 (*Trim16)* and valosin containing protein (*Vcp)*, engaged for the induction of lysophagy (Fig. 7L, M), and genes involved in the protein quality control system such as heat shock protein family B (small) member 8 (*Hspb8), Bag cochaperone (Bag1)*, and *Bag3* (Fig. 7N–P). Notably, we observed that, in presence of HTTex1-145Q, *Lgals3* and *Bag3* levels were significantly upregulated, thus suggesting the activation of a cellular response aimed at boosting autophagic clearance of muHTT and to activate LQC to preserve lysosomal integrity.

### TFEB and TFE3 sequestration affects lysosomal membrane integrity

It is known that lysosomal functions are altered in HD [55]. Based on the observation that *Lgals3* expression was increased in the presence of HTTex1-145Q, we assessed the role of lysosomes and LGALS3 in HD.

**Fig. 8:**
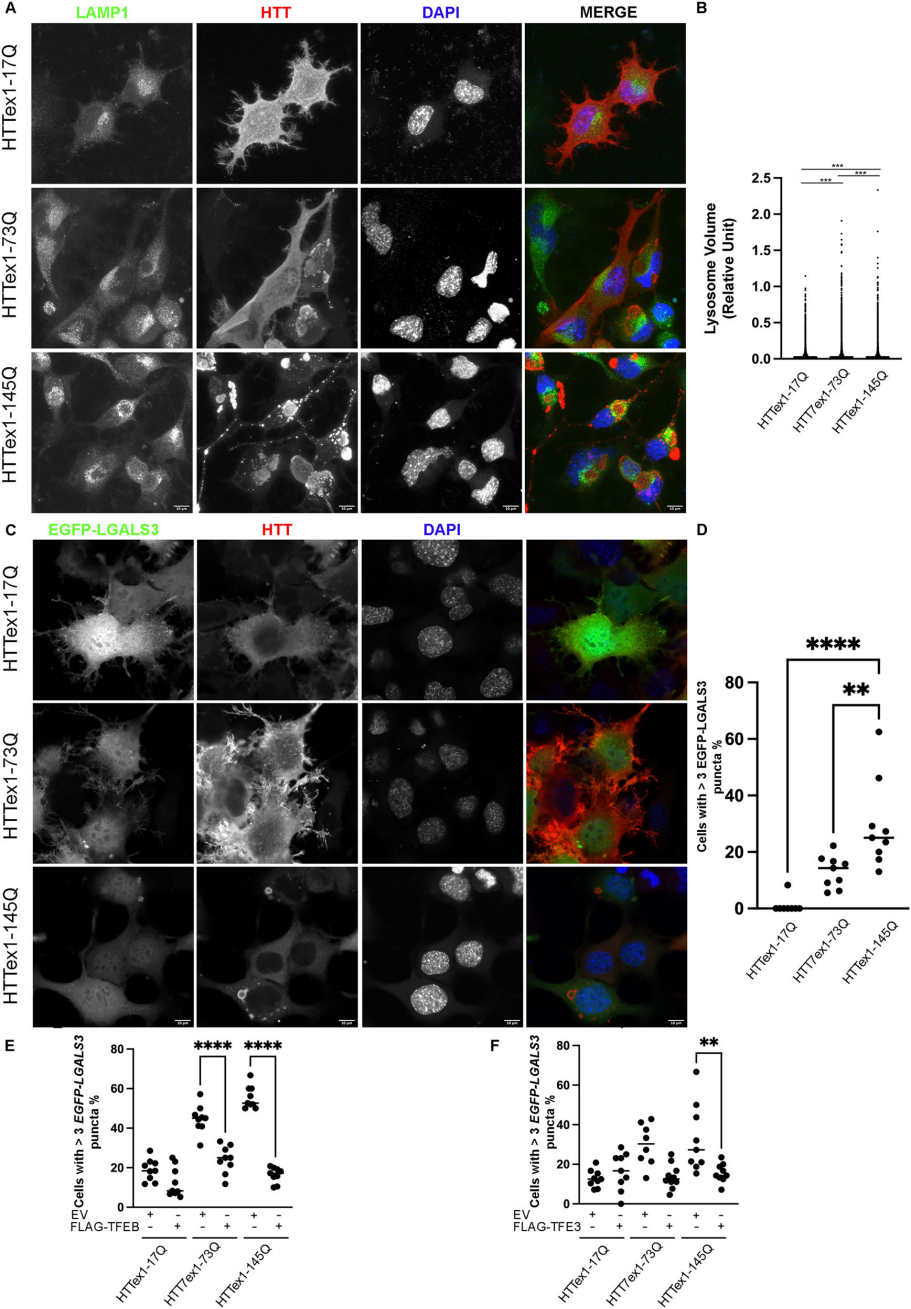
TFEB and TFE3 sequestration affects the LQC. **A**, **B** NSC-34 cells were transfected with wt or muHTT. (**A**) Representative images by confocal microscopy of double immunostaining with anti-LAMP1 (green) and anti-HTT antibody (red), nuclei were stained with DAPI (blue) (63X magnification). Scale bar: 10 μm. **B** the scatter dot blot represents the quantification of lysosome volume. The fields were randomly selected and at least 100 cells for each sample were analyzed (***p < 0.001, one-way ANOVA with Tukey’s test). **C**, **D** NSC-34 cells were co-transfected with wt or mutant HTT and EGFP-LGALS3. **C** Representative images by confocal microscopy of EGFP-LGALS3 (green) and IF staining with anti-HTT antibody (red), nuclei were stained with DAPI (blue) (63X magnification). Scale bar: 10 μm. **D** LGALS3 puncta assay. The bar graph represents the quantification of percentage of cells with >3 GFP-LGALS3 puncta. (** *p* < 0.005, **** *p* < 0.0001, one-way ANOVA with Tukey’s test). NSC-34 cells were co-transfected with EGFP-LGALS3, FLAG-TFEB (**E**), FLAG-TFE3 (**F**), or EV, and wt or muHTT. LGALS3 puncta assay was performed. The graphs with individual values represent the quantification of percentage of cells with >3 GFP-LGALS3 puncta (**p < 0.005, ****p < 0.0001, two-way ANOVA with Bonferroni’s test).

First, we examined whether muHTT affects lysosome size. Double IF analysis of HTT and LAMP-1 in combination with quantitative analysis with ARIVIS software were used to analyze endolysosomal vesicles. We noticed that the volume of LAMP-1 positive structures increased in cells expressing muHTT compared to those with wt HTT, and this increase was also correlated with the polyQ tract expansion (Fig. 8A, B for quantification). Altered lysosomal activity has been shown to be due to alterations in lysosomal membrane integrity. Thus, we investigated whether these lysosomal alterations were correlated with LMP events, caused by muHTT. We performed an LGALS3 puncta assay to evaluate lysosomal membrane integrity in presence of wt or muHTT. We expressed wt and muHTTs with the plasmid GFP-LGALS3, coding for GFP-tagged human LGALS3. In presence of HTTex1-17Q, GFP-LGALS3 was mostly diffused, meanwhile we observed an increased accumulation of LGALS3 puncta in the leaky lysosomes in muHTTs- expressing cells suggesting that the presence of muHTT concurs to LMP and the impairment of LQC (Fig. 8C, D for quantification).

Interestingly, we observed that both *TFEB* and *TFE3* overexpression reduced GFP-LGALS3 puncta caused by *muHTT* expression (Fig. 8E, F). More specifically, TFEB was capable of restoring lysosome integrity both in presence of HTTex1-73Q and of HTTex1-145Q. Conversely, *TFE3* overexpression was effective only on HTTex1-145Q (Fig. 8E, F), suggesting a specific involvement related to insolubility of muHTT aggregates.

## Discussion

As postmitotic cells, neurons are sensitive to the accumulation of misfolded proteins and damaged organelles; in this context, lysosomes contribute to the maintenance of neuronal homeostasis with their degradative and signaling functions. In HD, the autophagy-lysosomal pathway is impaired at multiple levels and lysosomal functionality is compromised. In the last decades, TFEB, the main regulator of autophagy, has been found to be dysregulated in HD [35, 56], and approaches aimed to activate the TFEB pathway, with exogenous *TFEB* expression or with pharmacological activation, facilitate muHTT clearance and ameliorate disease phenotype [53, 57, 58].

Our findings indicate that muHTT sequesters TFEB and TFE3 into inclusions, preventing or reducing their activities, a patho-mechanism that results in lysosomal damage. Interestingly, muHTT did not fully co-localize with LGALS3 puncta, suggesting that LMP is not directly caused by protein aggregates entrapped into lysosomes, but rather that the accumulation of damaged lysosomes might be caused by the depletion of TFEB and TFE3 in aggregates. The reduced TFEB/TFE3 bioavailability impairs the transcription of Coordinated Lysosomal Expression And Regulation genes, including those involved in lysosome homeostasis, thus preventing the activation of lysophagy and the clearance of damaged lysosomes. TFEB/TFE3 and lysosomes are also involved in Calcium homeostasis [59, 60], and downstream negative events (e.g., Calcium homeostasis dysregulation) in cells.

This might activate a vicious cycle in which cells, unable to trigger damaged lysosome clearance via lysophagy and to replace them via their biogenesis, become increasingly sensitive to muHTT inclusions, which in turn continue to accumulate in affected cells. In fact, we demonstrated that *TFEB* and *TFE3* overexpression restores a functional LQC system, leading to enhanced muHTT clearance and concomitantly to the clearance of damaged LGALS3-positive lysosomes, ultimately preventing or blocking the appearance of the neurotoxic vicious cycle described above.

Our data partially disagree with a previous study performed in a different HD mouse model expressing 140 CAGs, showing that the TFEB sequestration into muHTT inclusions is mediated by the PrLD, near the N-terminus of TFEB. However, TFE3, lacking this domain, did not co-aggregate with muHTT [35]. Our in vivo findings showed that both TFEB and TFE3 co-localize with muHTT aggregates in the striatum and cortex of HD mouse models expressing 190 CAGs. Moreover, in their in vitro experiments, the above-mentioned authors used the GFP-tagged variant of HTT, with a polyQ length of 74; instead, we used plasmids coding for two different polyQ lengths (73Q and 145Q) without the fluorescent tag. Beyond these technical differences that might partially explain the discrepancy of the results, we observed a different behavior between TFEB and TFE3 in presence of the two forms of muHTT. In fact, TFEB resulted to significantly co-aggregate with HTTex1-73Q and HTTex1-145Q (Figs. 3A, B, and 5C, G), instead TFE co-aggregated selectively with the HTTex1-145Q. Moreover, *TFEB* overexpression was capable of restoring lysosome integrity by reducing LGALS3 accumulation in presence of both muHTT forms; instead, *TFE3* overexpression acted only on HTTex1-145Q. Notably, *Tfe3* expression was upregulated in muHTT mice (Fig. 1F), while no variations were observed in the cell model (Fig. 7B), indicating that *Tfe3* might be induced in response to chronic exposure to muHTT. These results suggest, for the first time, that the two transcription factors may exert different activities in the regulation of the autophagy-lysosome pathway that can be evidenced in the case of HD pathogenesis, in particular in patients carrying expanded CAG tracts of different lengths, which might differently influence their behavior. The fact that these two transcriptional regulators of the autophagy-lysosome pathway may have different, still unrecognized functions is supported by the fact that homozygous mutations in the *Tfeb* gene are lethal in mice, while *Tfe*3 mutated mice are viable even if with an altered energy metabolism [61, 62]. This also reinforces the notion that, despite a large overlap in their functionality, TFEB and TFE3 also play independent and different functions potentially to the pathogenesis of other NDs. In our study, beyond these novel differences in activities, as expected, TFEB and TFE3 showed similarities in their ability to regulate lysosomal biogenesis and autophagy in response to different sources of cellular stress in multiple cell types. In HD, muHTT aggregation is polyQ length-dependent and its aggregation mechanisms are partially unclear; several hypotheses have been proposed, highlighting different states with oligomers formation and amorphous aggregates [63–67]; in addition, the increased length of the polyQ tract directly correlates with the nature of the inclusions, where the longer polyQ tracts give rise to larger and more insoluble inclusions. Our findings suggest that the PrLD mediates the sequestration of TFEB into muHTT inclusions, while in presence of the long and more insoluble polyQ tract with 145Q both TFEB and TFE3 co-aggregate with the mutant protein, suggesting that other mechanisms might be involved in this process.

We found that TFEB and TFE3 mislocalization causes lysosome defects and damages that cannot be repaired by lysophagy and affects cell viability. Reduced TFEB and TFE3 nuclear localization has been already reported in different NDs [68–70] and accumulating evidence has revealed that lysosomal damage and LQC impairment might contribute to the pathogenesis of these diseases. In fact, the cell-to-cell transmission of misfolded protein fibrils is mediated by their extracellular release and binding to the plasma membrane of neighboring cells, leading to intracellular uptake via endocytosis. These fibrils are delivered to lysosomes, where they cause damage and escape into the cytosol, accumulating in aggregates. To counteract these toxic events the cell attempts to activate lysophagy to clear damaged lysosomes. Interestingly, LGALS3 was found increased in the cerebrospinal fluid of Alzheimer disease patients and involved in the unconventional secretion of these spreading aggregates in Alzheimer and Parkinson disease [71–74]. In HD, high levels of LGALS3 have been found in plasma and brain of patients and mice. LGALS3 upregulation was observed in HD mice before the motor symptoms, in the microglia LGALS3 was found associated to damaged lysosomes and its suppression in microglia ameliorated the HD mice phenotype [36]. LGALS3 is emerging as a key factor for NDs for its intracellular role in lysosomal damage, but also for its functions linked to its secretion in the extracellular space. Many pieces of evidence suggest its detrimental role in neurodegeneration, even if a protective role of LGALS3 has been reported (reviewed in ref. [75]). LGALS3 mechanisms of action need further investigation but its pharmacological modulation might represent a valuable target for intervention for NDs. LGALS3 inhibitors have already been tested in metabolic and fibrotic diseases, and these approaches might be applied to NDs. 3′-bis-(4-aryltriazol-1-yl) thiodigalactoside (GB039, formerly named TD139), a synthetic small molecule that antagonizes LGALS3 activity by binding to the carbohydrate recognition domain, was effective in idiopathic pulmonary fibrosis and retinal degeneration [76, 77]. Pectins, plant cell wall polysaccharides, mostly obtained from citrus and apples, represent natural LGALS3 inhibitors [78, 79].

In summary, our experiments suggest that LQC impairment might contribute to HD. Indeed, the LGALS3 accumulation observed in HD cellular models due to TFEB and TFE3 sequestration by muHTT inclusions causes LMP and lysophagy impairment, in turn, influences LQC.

## Supplementary information

Figure S1

Figure S2

Table S1

Table S2

Unedited blot images

## Data Availability

Data supporting the findings of this study are available from the corresponding author upon request.
