# Supplementary figures and images for: Impairment of lysosomal quality control in Huntington disease
Source: Cell Death Dis. 2025 Oct 27;16(1):762. doi: 10.1038/s41419-025-08103-z (PMC12559425; doi:10.1038/s41419-025-08103-z)

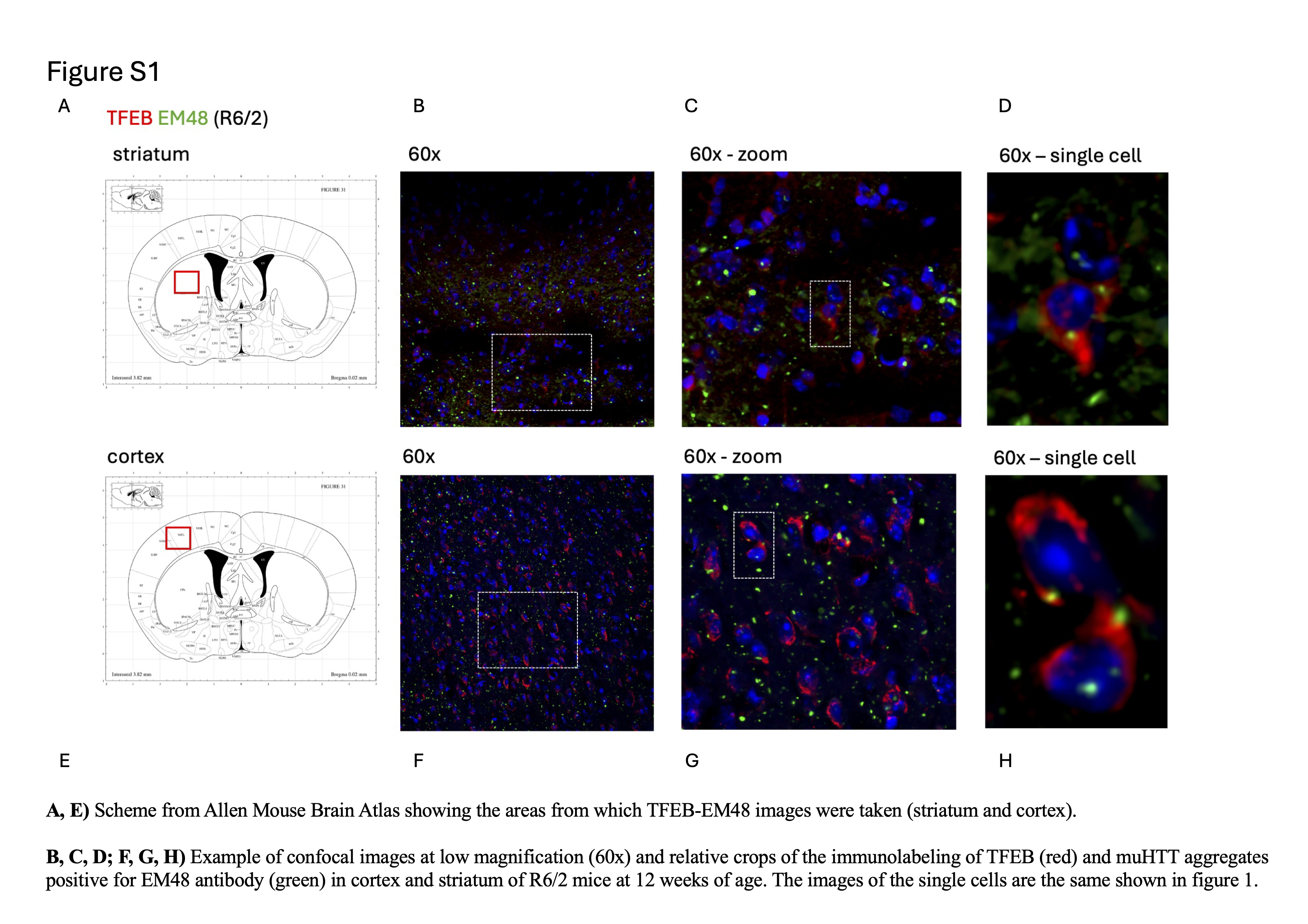

Supplement: Supplementary file 1 — Figure S1 [file 41419_2025_8103_MOESM1_ESM.tif]

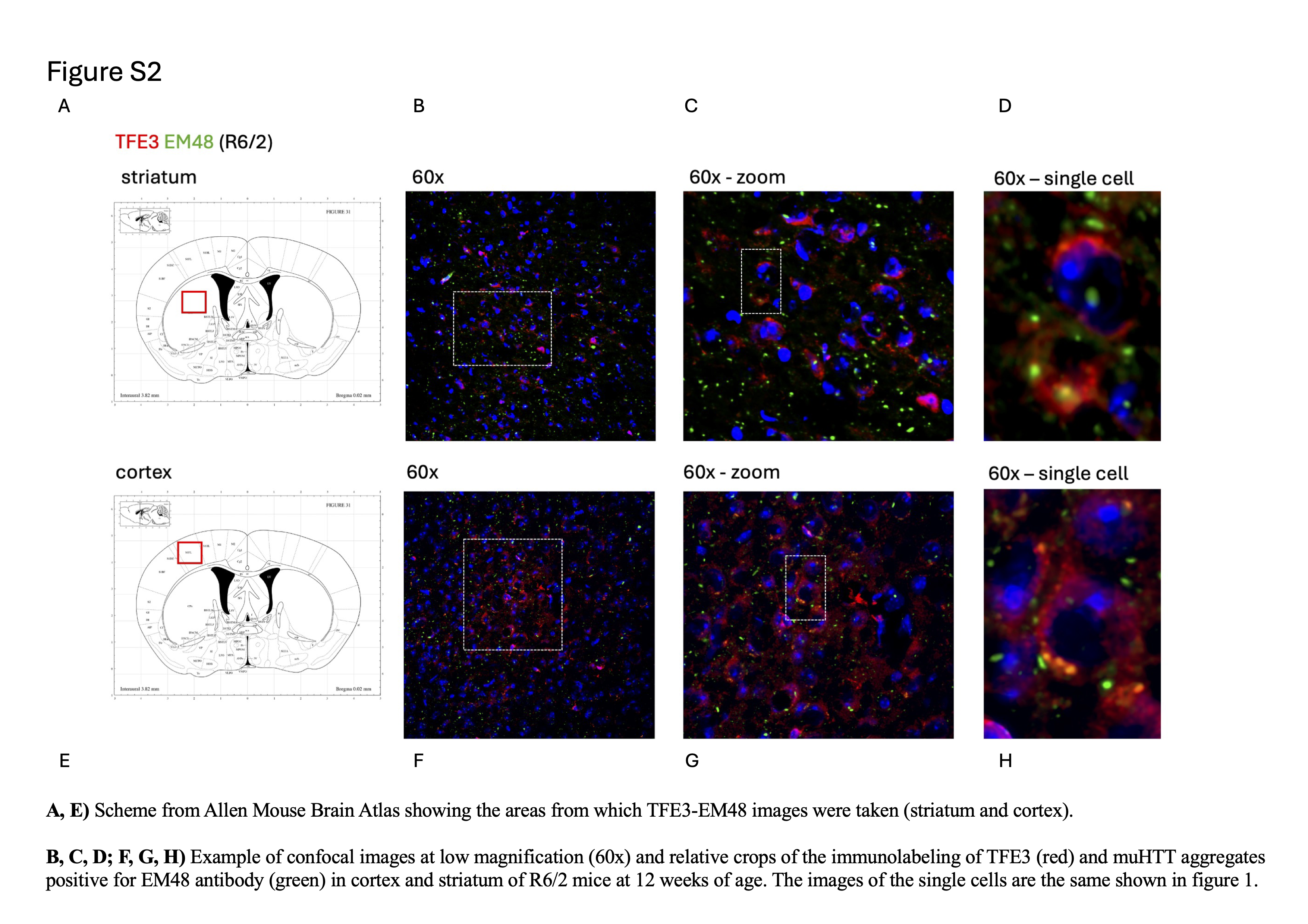

Supplement: Supplementary file 2 — Figure S2 [file 41419_2025_8103_MOESM2_ESM.tif]
