# Supplementary material for: Impairment of lysosomal quality control in Huntington disease
Source: Cell Death Dis. 2025 Oct 27;16(1):762. doi: 10.1038/s41419-025-08103-z (PMC12559425; doi:10.1038/s41419-025-08103-z)
Supplement: Supplementary file 5 — Unedited blot images [file 41419_2025_8103_MOESM5_ESM.pptx]

## Slide 1
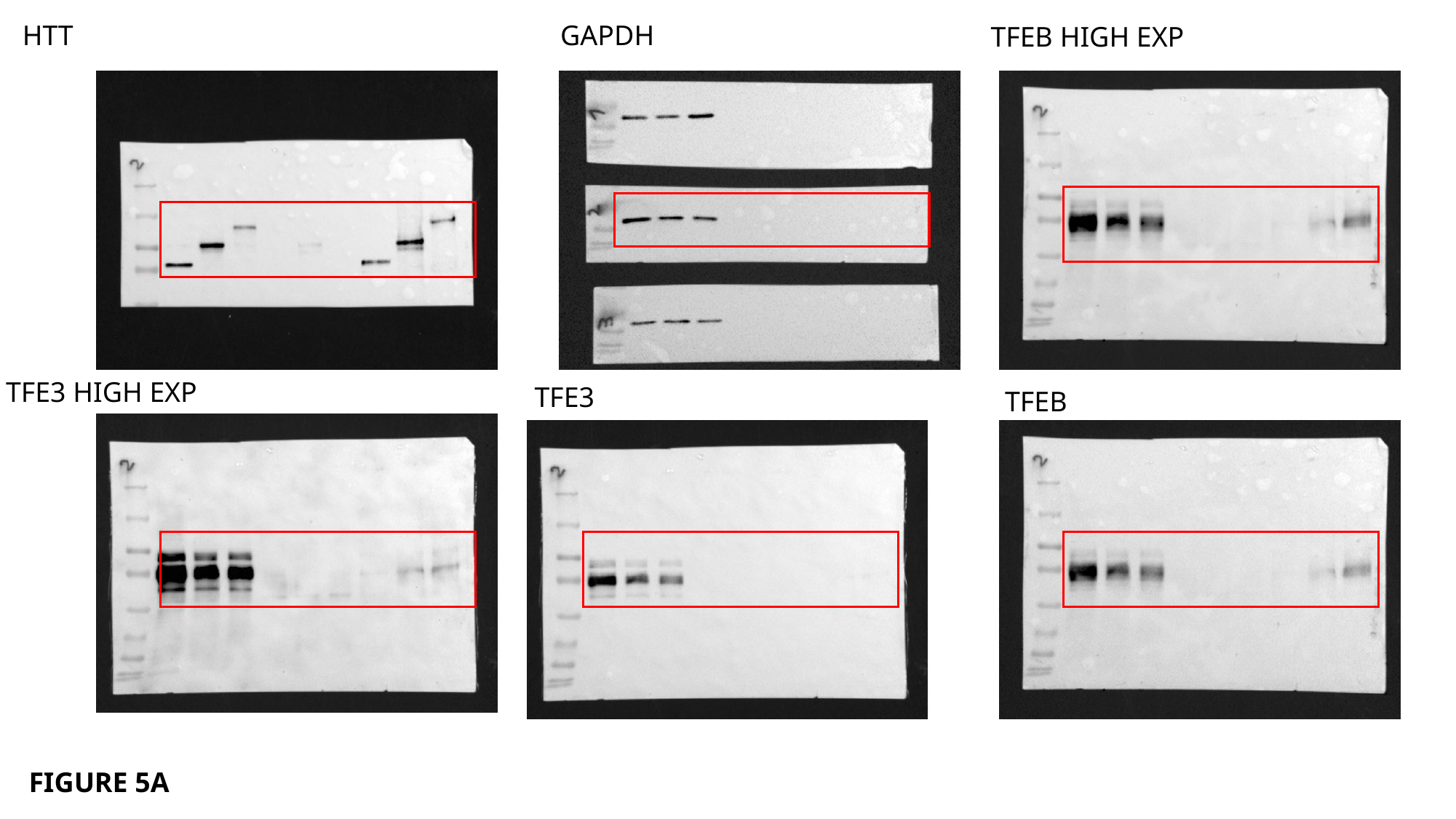

HTT
GAPDH
TFEB HIGH EXP
#
TFE3 HIGH EXP
TFE3
TFEB
FIGURE 5A

## Slide 2
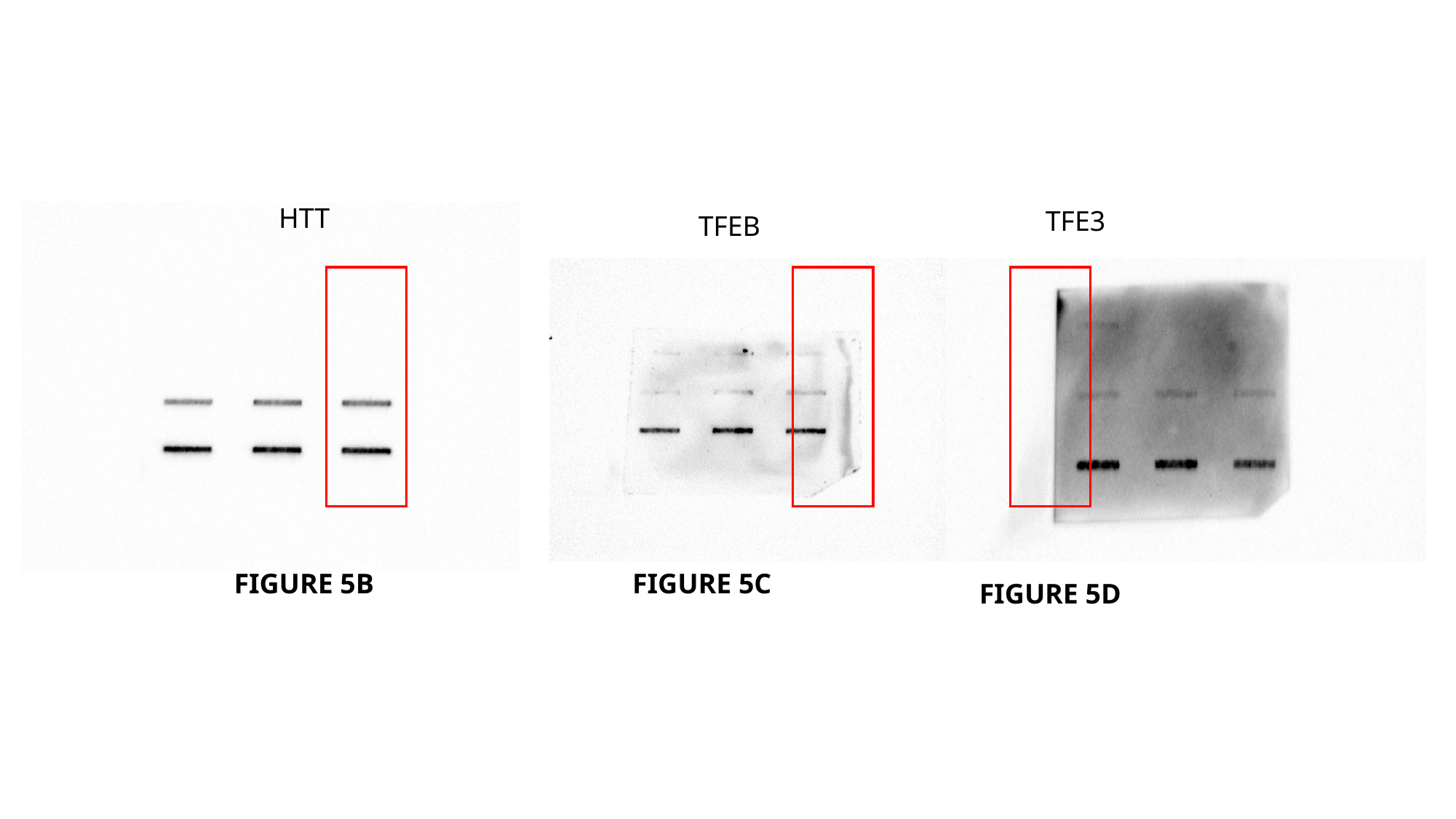

HTT
TFE3
TFEB
FIGURE 5B
FIGURE 5C
FIGURE 5D

## Slide 3
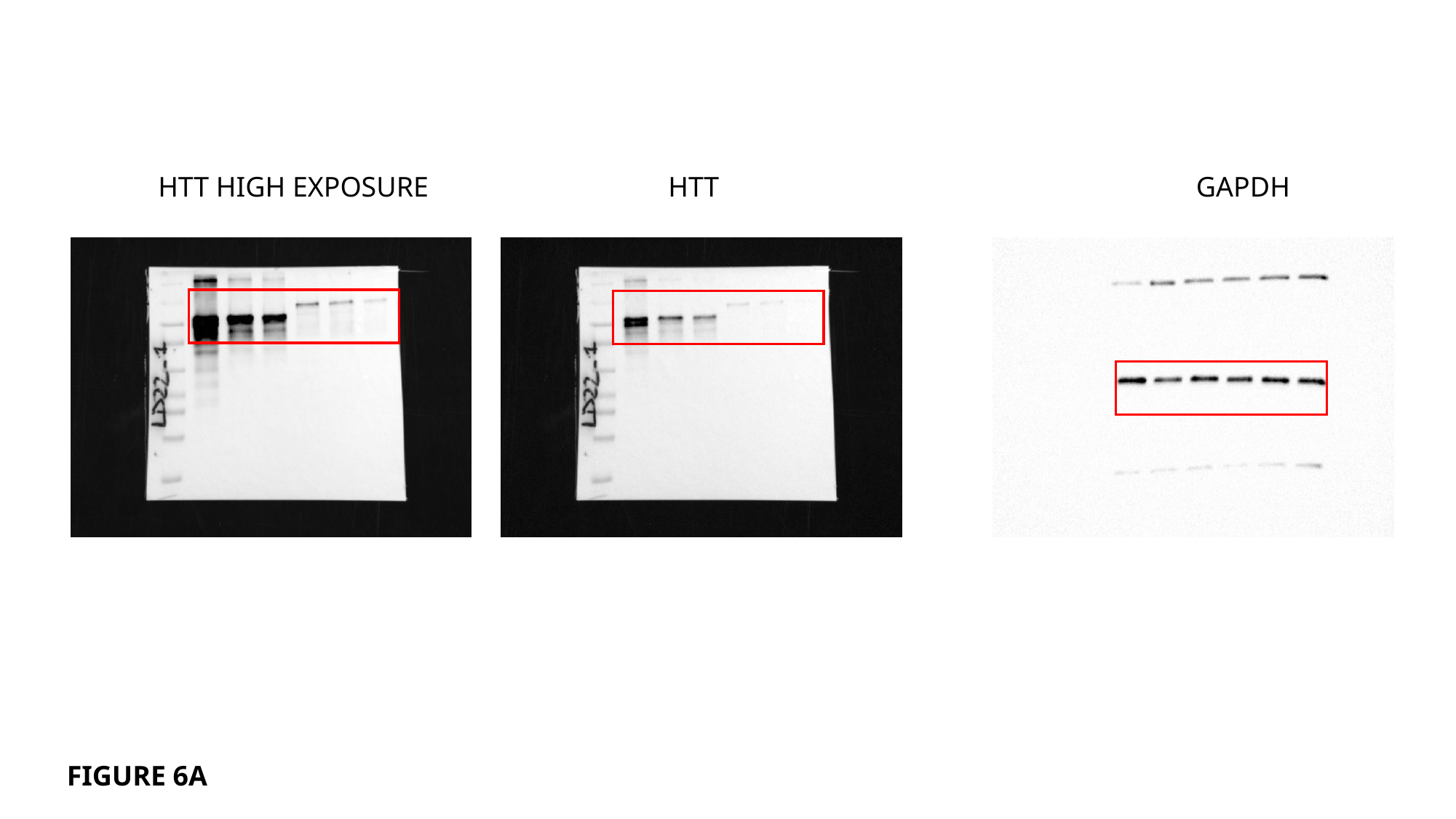

#
HTT HIGH EXPOSURE
HTT
GAPDH
FIGURE 6A

## Slide 4
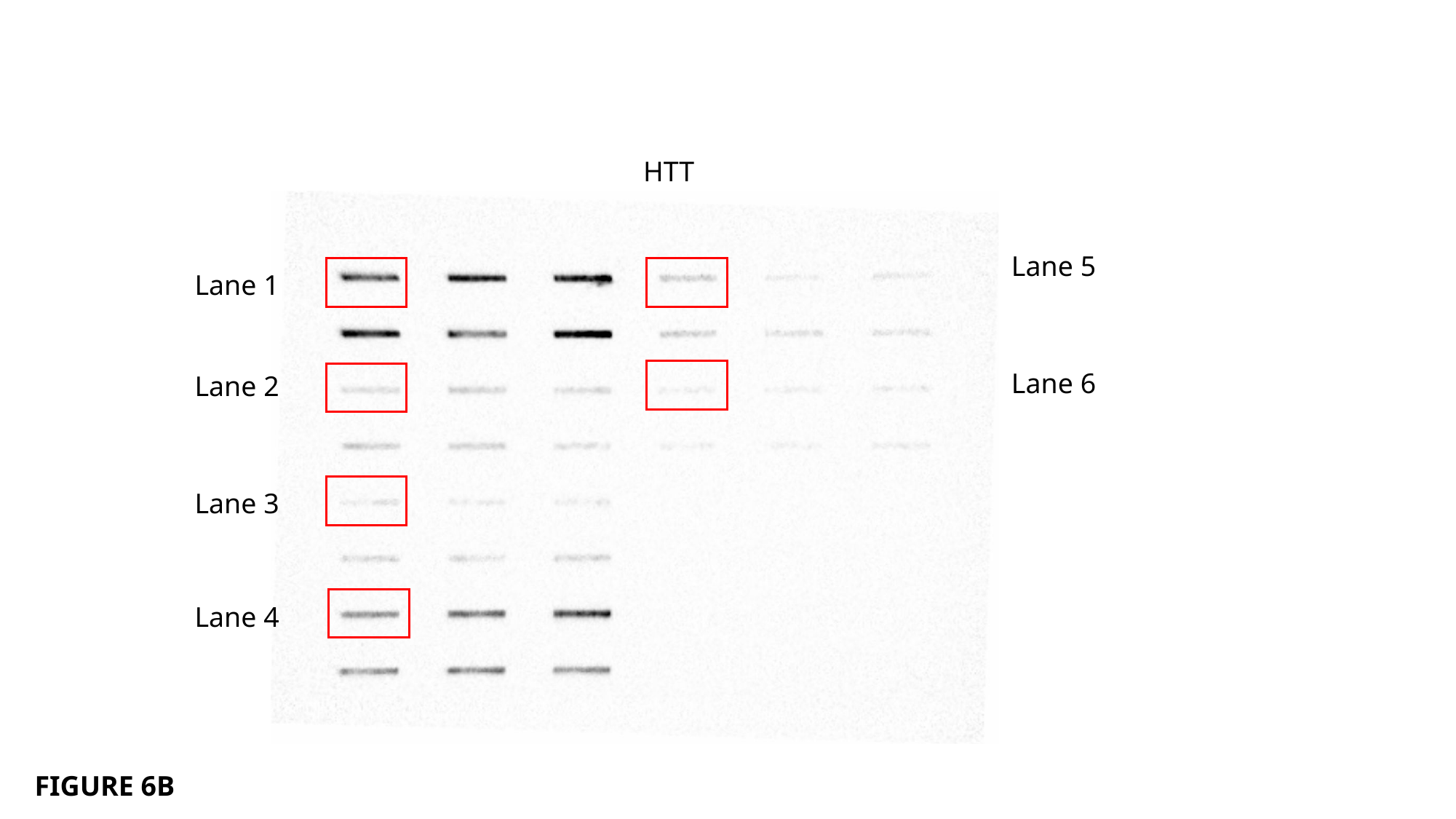

HTT
Lane 5
Lane 1
Lane 6
Lane 2
Lane 3
Lane 4
FIGURE 6B

## Slide 5
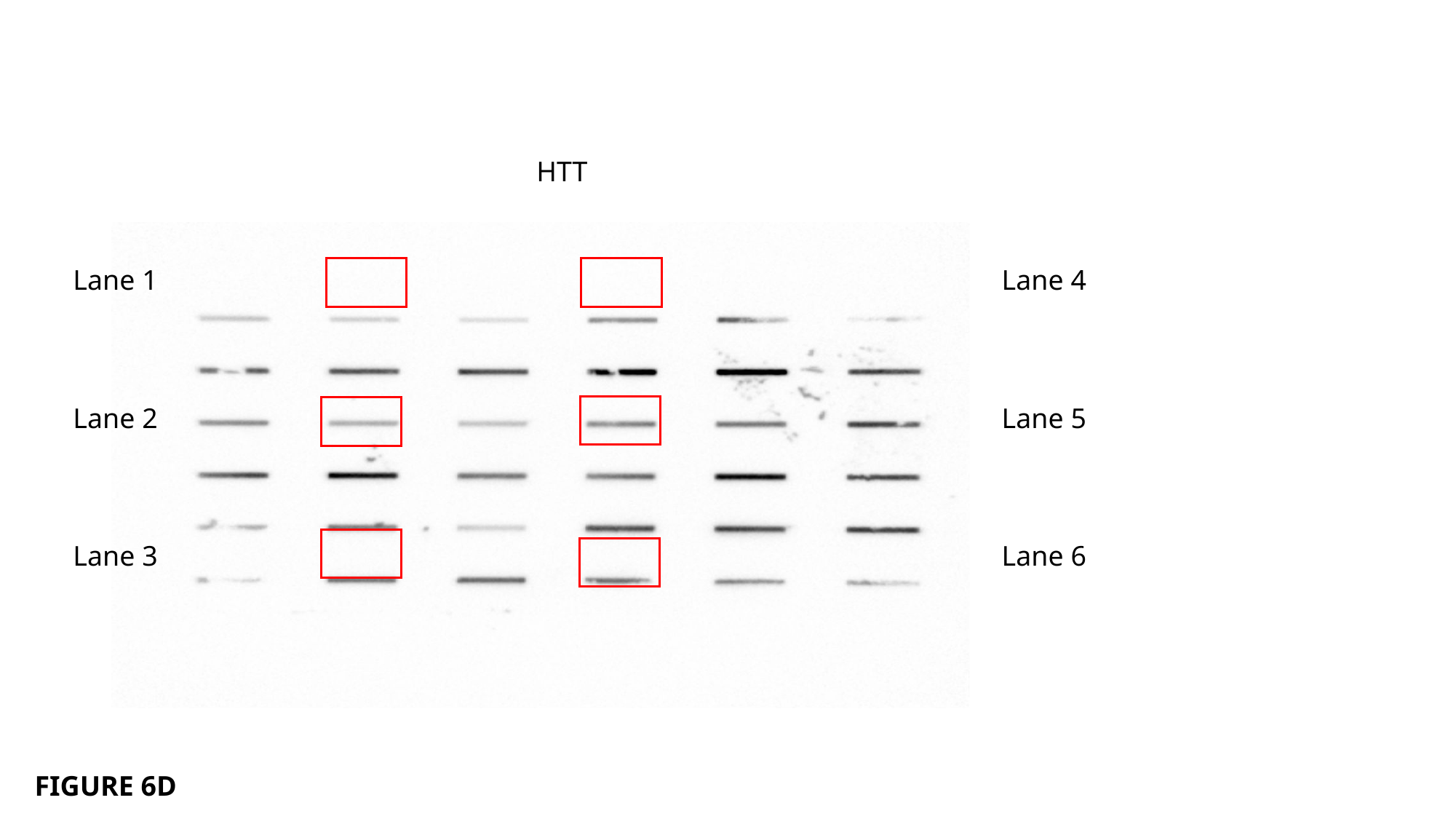

HTT
Lane 1
Lane 4
Lane 2
Lane 5
Lane 3
Lane 6
FIGURE 6D
